# Revisited Cyclophosphamide in the Treatment of Lupus Nephritis

**DOI:** 10.1155/2022/8345737

**Published:** 2022-05-26

**Authors:** Xiao-ying Quan, Hao-tao Chen, Si-qin Liang, Chen Yang, Cui-wei Yao, Yong-zhi Xu, Hua-feng Liu, Ning An

**Affiliations:** ^1^Key Laboratory of Prevention and Management of Chronic Kidney Disease of Zhanjiang City, Institute of Nephrology, Affiliated Hospital of Guangdong Medical University, Zhanjiang, Guangdong 524001, China; ^2^Guangdong Provincial Key Laboratory of Autophagy and Major Chronic Non-Communicable Diseases, Zhanjiang, Guangdong 524001, China

## Abstract

Lupus nephritis (LN) is the most common serious complication of systemic lupus erythematosus (SLE). The pathogenesis of LN is complex, and the majority causes of LN are the renal deposition of circulating or/and in situ-formed immune complexes. These immune complexes trigger glomerular and tubulointerstitial inflammation, which finally leads to proteinuria and loss of renal function. Despite the emergence of new biological agents, cyclophosphamide (CY), an alkylating agent, is still the first-line drug widely used to treat patients with severe LN. In this review, we outline the application history, molecular structure, and pharmacokinetics of CY in the treatment of LN. We also detail its latest known immunopharmacological mechanisms, with a focus on supplemental regulation and inhibition of CD4 and CD8 positive T cells, differences in the use of various guidelines, and the combination with other drugs. The side effects of CY are also mentioned in this review.

## 1. Introduction

Systemic lupus erythematosus (SLE) is an autoimmune disease involving multiple organs [1]. It is more common in women of childbearing age around 15 to 35 years [2]. The patient ratio of male to female is between 1 : 7 and 1 : 10, the incidence rate is about 7/10,000, and the female prevalence rate is 11.3/10,000 [3]. Light microscopy of renal biopsy samples shows renal damage in approximately 90% of patients with SLE [4]. Immunofluorescence and electron microscopy reveal renal lesions mainly associated with the glomerular and tubulointerstitial deposition of immune complexes [5, 6]. LN is a major determinant in SLE prognosis and once end-stage renal disease (ESRD) has been established, the disease markedly worsens [7].

The principles of LN treatment include immunosuppressive and symptomatic regimens for related manifestations and complications [8]. After decades of continuous discussion, great progress has been made in the immunosuppressive treatment of LN. The intensity of immunosuppressive therapy should be determined according to clinical and renal pathology [9–11]. Cyclophosphamide (CY), a commonly used immunosuppressant in the clinic, is a nonspecific alkylating agent, which mainly acts on the proliferative cells. CY inhibits the proliferation of T and B lymphocytes and inhibits the response of lymphoblasts to antigen stimulation in patients. It is considered the first choice for LN treatment, especially for severe LN [12]. Application based on glucocorticoid therapy has been suggested. The combination of CY and glucocorticoid can be more effective in prevention of doubling serum creatinine level than glucocorticoids alone [13]. CY pulse therapy is almost used in the stage of induction remission in LN treatment. However, CY can cause certain side effects, such as gastrointestinal reactions, nausea, and vomiting. Some patients may be unable to tolerate serious gastrointestinal reactions, while treatment in others may be stopped due to infection onset, liver damage, bone marrow suppression, and leukopenia. Gonadal suppression will also make young people with reproductive needs refuse the drug [14, 15]. How to minimize side effects has become an urgent problem to be solved. In this study, we review the mechanism, clinical application, and side effects of CY-induced remission in the treatment of LN.

## 2. Pharmacokinetics of CY

**Figure 1:**
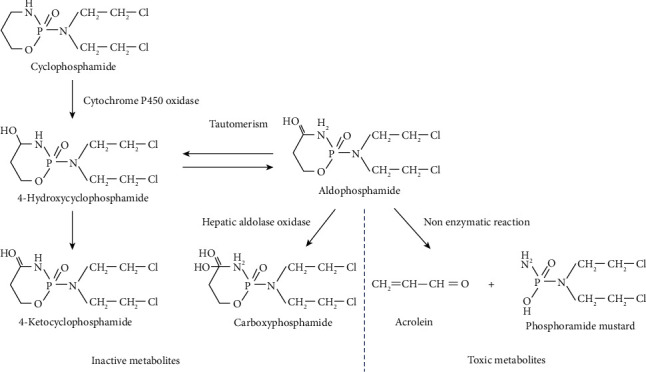
Metabolism of CY.

CY is an alkylating agent which has no effect in vitro and works only after it enters the body and is activated. CY is oxidized by cytochrome P450 oxidase in the liver to produce 4-hydroxycyclophosphamide. 4-Hydroxycyclophosphamide can be further oxidized and metabolized into nontoxic 4-ketocyclophosphamide or be formed into aldehyde compounds such as aldophosphamide through tautomerism. One part of aldehyde compounds is transported to the blood circulation and then further oxidized in the liver to form nontoxic carboxylic acid compounds. One part of aldehyde compounds is transported to the normal tissues which cannot carry out the above metabolism and can only undergo nonenzymatic reaction like *β*-eliminate to form into acrolein and phosphoramide mustard (Figure 1). Phosphoramide mustard is a key component of cytotoxicity, which alkylates DNA and interferes with its replication by forming crosslinks, thereby arresting the cell cycle, and acting against cell proliferation. Alkylating agents are the main acting metabolites while acrolein is a byproduct that increases toxic side effects. So, only neutralizing acrolein may reduce toxicity and increase efficiency [16].

CY is a cell cycle-nonspecific drug that mainly acts during the S phase and blocks cell division before the G2 phase. It has no obvious effect on resting cells but a strong effect on proliferating cells. At the same time, phosphoramide mustard can also increase the expression of proapoptotic mRNA and inhibit the expression of antiapoptotic mRNA to promote cell apoptosis [17].

The distribution of CY accords with the first-order atrioventricular model. The distribution capacity of CY is about 30 to 50 L. In oral administration, CY is rapidly absorbed and peaks after 1 to 3 hours with a bioavailability of about 85% to 100% [17]. The half-life of CY after intravenous injection is between 4 and 6 hours, which is shorter in children and adolescents compared with adults. Within 48 hours, 50%-70% of CY can be excreted from the kidney, 68% are metabolites, and 32% is a prototype. The main metabolites of CY excreted by urine include carboxycyclophosphamide and phosphoramide mustard. Thirty percent of the active dose is excreted via the urine; so, it shows certain stimulation to the kidney and bladder. Moreover, acrolein, a CY metabolite, has a direct toxic effect on bladder epithelial cells [18]. Therefore, it is necessary to be alert to the toxicity of CY to the urinary system [19]. The renal clearance of CY is related to urinary flow rate; so, hydration therapy can accelerate drug excretion.

It is difficult to detect the plasma concentration of CY metabolites. Recently, the conventional detection methods include HPLC, GC-MS/MS, or LC-MS/MS. Most detection methods only detect one or two compounds, and most of them determine the blood concentration after routine dose or low-dose CY [20–22]. It has been shown that the polymorphic CY2B6 is an important enzyme for the bioactivation of CY. Moreover, CY-inducing agents targeting CY2B6 might be used to enhance drug activation and therapeutic efficacy [23]. Some drugs such as barbiturates, corticosteroids, allopurinol, and chloramphenicol can affect the activity of liver microsomal enzymes and the metabolism, toxicity, and activity of CY.

## 3. Immunopharmacological Mechanism of CY in LN Treatment

Cell apoptosis, loss of self-tolerance, and dysfunction of the cell clearance system in patients with systemic lupus erythematosus lead to the accumulation of a variety of autoantibodies and free nucleosomes, resulting in the formation and deposition of many immune complexes (IC) in the kidney [24]. IC deposition stimulates the complement cascade reaction, promotes the proliferation and activation of glomerular mesangial cells, and releases a variety of inflammatory factors that leading to glomerular diseases [25]. In addition, in situ IC deposition is also found in the basement membrane, vascular wall, and subendothelium. In vitro experiments have shown that the structure and composition of type IV collagen on the glomerular basement membrane of LN patients changed, which not only enhances the affinity for DNA but also exposes new antigens on the basement membrane, facilitating the binding of autoantibody [26]. It can be inferred that the renal in situ IC of lupus patients first binds to the DNA on the glomerular basement membrane and then absorbs the anti-DNA antibody in the blood to form IC locally in the kidney. The deposition of IC in the kidney, on the one hand, is due to the massive production of IC in the body; on the other hand, IC clearance is hindered, as seen by the inhibition of complement mediated immunoprecipitation and weakening of immune adhesion.

CY can exert extensive toxic effects on lymphocytes in immune organs such as the thymus and spleen. CY-induced remission in LN can be related to the extensive regulation on the humoral immunity. CY significantly reduces the number of B cells and inhibits the production of autoantibodies. CY can reduce the production of IgG and IgM, but IgE production increases, suggesting a certain CY selectivity in inhibiting antibody production [27]. Simultaneously, CY reduces the function of B cells by inhibiting helper T cells. CY reduces the absolute number of T lymphocytes and B lymphocytes, especially for B lymphocytes in the early stage. In rodents, CY can mediate lymphocyte loss, which is mainly manifested in the reduction of lymphoid follicles and germinal centers [28, 29]. Generally, the loss reaches the peak three days after cyclophosphamide treatment, and the cell density can return to the original level seven days later [28, 30]. After administration of CY, the number of B cells in the thymus and spleen of mice consumes rapidly within 24 hours, and the number of T cells also decreases delayed. Finally, it decreases to the lowest within 2 to 7 days after cyclophosphamide treatment [31, 32]. B depletion induced by CY is earlier, which proved that B cells are more sensitive to CY than T cells. Sensitivity of lymphocyte subsets to CY is different. It has been suggested that the sensitivity of lymphocytes to CY can be arranged in order as follows: B cells > suppressor T cells > helper T cells > cytotoxic T cells [33].

The inhibitory effect of CY on cellular immunity is dose-dependent. A high-dose CY can cause damage to the immune system, and low-dose CY has immune enhancement effect. Low-dose CY mainly inhibits the proliferation and function of CD8 cells but has no significant effect on CD4 cells. With the increase in CY dose, the inhibitory effect on CD4 cells becomes stronger, but the effect on CD8 cells does not change significantly. So, CD4 T lymphocyte subsets may be more sensitive than CD8 T lymphocyte subsets. CD8 cells recover the fastest after CY treatment, and the recovery of B cells gradually slows down, while CD4 cells recover to the original level after 4 months [34]. CY not only suppresses the proliferation of effector T cells but also abrogates the expansion of CD4^+^ Foxp3^+^ regulatory T cells [35, 36]. However, when combined with IL-2, CY administration allows CD4 T cells transferring into regulatory T cells to alleviate autoimmune disease [37].

However, these findings about CY inhibition of T cells were challenged recently. Fassbinder et al. [38] have found that induction therapy with CY was related with an increase in circulating CD8 effector T cells and plasmacytoid dendritic cells. Gabcova et al. [39] have reported that Euro-Lupus low-dose IV CY increased the ratio of CD8 T cells, regulatory T cells, neutrophils, and monocyte subsets when compared to health control. More research is needed to explore the accurate effects of CY on other immune cells.

## 4. Clinical Use of CY in Induction Remission in LN

It is important to timely recognize and treat kidney disease, because early treatment response is related to better prognosis. At present, there is a lack of research on the treatment of type III + V or IV + V LN, and the guidelines only suggest that the induction treatment of this kind of patients is the same as that of type III or IV. Therefore, for newly diagnosed proliferative LN (type III, type IV, type III + V, or type IV + V), the goal of treatment is to achieve rapid renal remission by induction therapy, avoid chronic kidney damage by maintenance therapy, and minimize treatment-related toxic side effects [40]. The treatment process of LN includes two stages: induction of remission and maintenance treatment. The initial induction of remission is the key stage in the treatment of severe LN patients, which generally lasts for 3-6 months. At this stage, glucocorticoids and immunosuppressive drugs (such as CY, azathioprine, mycophenolate mofetil, cyclosporine A, and tacrolimus) can be combined. If the condition is stable and reaches partial or complete remission, maintenance treatment can be implemented [41]. If the treatment response is poor, other alternatives to initial-induced remission therapy can be selected. The course of the maintenance treatment is 6–24 months. Patients with complete remission can gradually reduce or even stop treatment within one year, while patients with partial remission need to continue maintenance treatment [42].

CY has been used in the treatment of LN for more than 50 years. It is the most used first-line drug for the treatment of LN, especially critical LN. In 1986, Austin published the results of a large, randomized study affirming the role of intravenous infusion of CY as an induced remission in LN treatment. Follow-up studies in the National Institutes of Health (NIH) [43–45] found that the combination of CY and glucocorticoid could be more effective in improving the prognosis of patients with renal disease than glucocorticoid alone. Thus, it laid a foundation for the use of CY as an important drug for SLE and determined the NIH standard. The induction phase is the intravenous injection of CY once a month 6 or 7 times in total. Furthermore, 500 to 1000 mg/m^2^ (body surface area) of CY or combined with a venous drip of methylprednisolone or daily oral administration of hormones has been considered the gold standard for inducing renal remission and preventing renal flares. However, long-term use of high-dose CY has resulted in a series of serious adverse reactions, especially irreversible reproductive toxicity, and severe infection [46].

To reduce the total exposure and toxic response to CY, therapies for low-dose intermittent intravenous infusion of CY emerged. To compare the efficacy and side effects of CY, The Euro-Lupus Nephritis Trial conducted a multicenter, randomized, and open-ended controlled study on 90 cases of diffuse proliferative LN in 18 hospitals in 9 countries. The results showed that the cumulative probability of achieving renal remission and renal flare of low-dose CY-induced remission therapy (500 mg, once every 2 weeks, 6 consecutive times) were similar to that of traditional treatment regimen (0.75~1.0 g/m^2^, once a month, treatment for 6 months) (HR 1.26, 95% CI 0.72–2.21; *P* = 0.36), but the adverse reactions such as infection, menstrual disorder, amenorrhea, and bone marrow suppression were significantly reduced [47]. In recent years, several international organizations including Kidney Disease Global Outcomes (KDIGO), American College of Rheumatology (ACR), and The European Alliance of Associations for Rheumatology (EULAR) have successively introduced treatment guidelines for LN. KDIGO [48] and ACR [49] guidelines recommended high-dose intravenous CY regimen (0.75~1.0 g/m^2^, once a month, treatment for 6 months) while EULAR [50] guidelines only recommended low-dose intravenous CY (500 mg, once every 2 weeks, 6 consecutive times, the total amount does not exceed 3 g within 3 months) as the first choice (Table 1). CY is cheap and easy to administer compared with other immunosuppressants. This regimen remains the best choice for some poor areas. Compared with intermittent intravenous CY, daily oral CY is well tolerated, but the risk of hemorrhagic cystitis is increased. IV CY rarely produces hemorrhagic cystitis, but gastrointestinal symptoms are more serious.

Low dose prednisone (≤10 mg/d) combined with azathioprine (AZA) or mycophenolate mofetil (MMF) maintenance therapy is suggested be applied in type III/IV LN after induction remission in three guidelines. ALMS trial is a randomized, double-blind, double simulation, and placebo-controlled study, which shows that the treatment failure rate, the cumulative probability of renal flare, doubling of serum creatinine level, incidence of ESRD, and serious side effects in MMF group are lower than those in AZA [51]. However, international multicenter clinical studies show that the recurrence rate of MMF maintenance treatment is lower than that of AZA, but the difference in long-term prognosis is not clear [52]. Therefore, whether MMF or AZA is preferred is not clear in various guidelines, and the best course of AZA or MMF maintenance treatment is the most controversial. KDIGO guidelines suggest that the treatment should be continued for 3 years after complete remission, and then the immunosuppressant should be reduced. EULAR guidelines recommend that it should be maintained for at least 3 years unless there is a serious adverse drug reaction, and it should be maintained for as long as possible.

The clinical manifestations, pathological injury, treatment response, and prognosis of LN vary among different races with more patients in the nonwhite, non-Asian group responding to MMF than to CY. The Aspreva Lupus Management Study [53] showed that MMF was better than intravenous CY in blacks, Hispanics, and Latin Americans, but CY was better in Asians. Another study that included Caucasians, Asians, and African Americans at the same time found that the response of MMF and CY treatment was similar in Caucasians and Asians, and the remission rate of MMF was higher only in African Americans [54]. The above research showed that there is a large gap in the response of different ethnic groups to treatment.

There are many therapeutic drugs against LN immune tissue damage, from the earliest glucocorticoid, CY, mycophenolate mofetil (MMF), tacrolimus, and anti-human CD20 monoclonal antibody. All immunosuppressants have certain side effects, but the incidence is different. Clinicians need to carefully consider the choice of immunosuppressants its dose and its drug combinations. Pathological morphology and occurrence mechanism are the most important references for drug use [55]. High doses of hormone (oral prednisone 1 mg/kg) primarily block the effects of NF-*κ*B and inhibit various inflammatory factors. CY, azathioprine, MMF, and tacrolimus all play an important role in inhibiting lymphocyte proliferation. However, each drug has its own characteristics and adverse reactions. MMF inhibits lesions of vascular endothelial cells, while tacrolimus inhibits interleukin-10 and affects B lymphocyte function. The advantage of MMF is its ability to control vascular inflammatory lesions and tacrolimus is propitious to the control of membranous lesions. Therefore, drugs are often applied to the treatment of various lesions at different therapeutic action points. The treatment method should be adjusted according to the pathological morphology and pathogenesis of tissue lesions. A large, randomized study randomly assigned 370 patients with classes III through V lupus nephritis to open-label MMF (target dosage 3 g/d) or CY (0.5 to 1.0 g/m^2^ in monthly pulses) did not detect a significantly different response rate between the two groups: 104 (56.2%) of 185 patients responded to MMF compared with 98 (53.0%) of 185 to IV CY [53]. Another randomized, open-label, parallel control, and noninferiority study found tacrolimus was noninferior to IV CY for LN response at week 24. There was a complete or partial response rate of 83.0% (117 of 141 patients) in the tacrolimus group and 75.0% (93 of 124 patients) in the IV CY group [56]. Results of representative studies of cyclophosphamide treatment compared to MMF or calcineurin inhibitors in patients with lupus nephritis were summarized in Table 2.

The current traditional treatment regimen for LN is glucocorticoid combined with CY. But in patients with refractory LN, when the traditional treatment regimen is ineffective, a switch of induction treatment after six months, from CY to MMF, or vice versa is suggested [62]. However, heterogeneity was a significant feature of LN. Multiple parts of the immune system participated in systemic and renal autoimmunity at the same time. The effect of treating refractory LN by intervening in a single way or consuming a single cell type is not satisfactory. Bao et al. first put forward the concept of multitarget therapy in the world. Combining immunosuppressants with different targets of action and reducing the dose of each immunosuppressant at the same time not only ensured the effect of drugs but also reduced the adverse reactions [63]. CNIs have been used as part of a multitarget approach to treat LN, added to a regimen of MMF and corticosteroids. Multitarget therapy comprising corticosteroids, tacrolimus, and MMF has been investigated in a prospective randomized open label trial for the induction treatment of LN and has been shown to be superior to intravenous CY in induction of remission at 6 months [64]. More recently, B cell-targeted therapy with rituximab has shown high remission rates in patients with refractory LN. Rituximab has shown encouraging results in the treatment of refractory LN, especially in class III and IV LN and to a lower extent in mixed classes and membranous LN [65]. Additional therapeutic options are emerging, such as other biologics, plasma exchange, immunoadsorption, calcineurin inhibitors, or eventually stem cell transplantation.

## 5. Side Effects of CY

### 5.1. Myelosuppression

CY can cause a transient decrease in leukocyte and granulocyte counts. In severe cases, a significant leukocyte and granulocyte deficiency could occur. The decrease in leukocytes caused by CY is dynamic, as leukocytes decrease significantly in 7 to 14 days and are recovered in 3 to 4 weeks. Therefore, the hemogram changes must be monitored within 7 to14 days, and CY should not be administered to patients with absolute neutrophil count ≤ 1500 per microliter and/or platelet count < 50 000 per microliter, which can avoid severe bone marrow suppression [66]. Oxidative stress is the major mechanism of myelosuppression caused by CY. Particularly, as one major toxic byproduct of CY metabolism, acrolein reacts with glutathione to deplete the cellular antioxidant defense system of peripheral blood and bone marrow [67].

### 5.2. Infection

Infection often occurs in patients with LN, resulting from the comprehensive effect of basic immune deficiency and immunosuppressants. Patients administrated by CY are susceptible to infection resulting from myelosuppression. Patients treated with IV CY are at risk of infection within a limited period of self-limited cytopenia, while those who received daily oral CY had a longer risk period of infection if they had chronic leucopenia, that may be one of the reasons for the different infection rates between IV CY and oral CY [68, 69]. The number of white blood cells in CY treatment above 2 × 10^9^/L indicates a low risk of serious infection. Infective pathogens include bacteria and viruses and fungi, and infection especially happens in the respiratory tract, digestive tract, and urogenital system. The incidence of herpes zoster is significantly higher when combined with hormone therapy than immunosuppressants alone [70]. To prevent serious infection, antibiotics can be used in combination, but antibiotic abuse must be avoided.

### 5.3. Gonadal Toxicity

The gonadal toxicity of CY can cause menstrual disorders and amenorrhea in female and oligospermia and azoospermia in male. Boumpas [71] reported that the risk of amenorrhea in patients with CY treatment is depended on age and dosage and during time of treatment. There is little risk of amenorrhea within 6 months in the patient younger than 25 years old, while the risk of amenorrhea is significantly increased in the patients older than 30 after receiving high-dose CY treatment. The toxic effect caused by CY is increasing with the delay of the age at which CY is taken. The cumulative dose of CY causing gonadal damage was 400 mg/kg in women of puberty and 200 mg/kg in women of reproductive age. Gonadal toxicity induced by low-dose CY is significantly lower than that caused by conventional dosage of oral CY. The lower peak concentration of CY may not play a major role in gonadal toxicity [72]. High-dose and long-term use of CY in men can cause sperm DNA damage and oligospermia. Before clinical application of CY, patients that may have a need for fertility or lactation must be informed of possible risks. Gonadotropin-releasing hormone inhibits the release of follicle-stimulating hormone and pituitary luteinizing hormone [73, 74], inhibits follicular mitosis, and protects ovarian reserve function. Now, the development and use of drugs promoting gonadotropin-releasing hormone have become a hot topic in the research of reducing reproductive toxicity caused by CY.

### 5.4. Bladder Toxicity

Hemorrhagic cystitis is another common complication in patients with CY treatment. Compared with the 17% incidence of oral administration of CY, the risk period of bladder damage induced by IV CY is shorter, mainly within 36 hours after administration [75]. The risk of injury is reduced due to hydration and emptying. However, the risk of bladder stimulation and infection is sustained in patients treated with a conventional oral dosage of CY. Controlling the cumulative dose and shortening the course are the main means to prevent hemorrhagic cystitis. In addition, strengthening diuresis and hydration and avoiding bedtime administration have been proven to reduce the hemorrhagic cystitis risk. Acrolein, a metabolite of CY, is toxic to the bladder epithelium and may be the cause of several bladder complications [76]. Recently, chloroacetaldehyde, another metabolite of CY, is considered to cause more severe damage of urothelial cells by increasing reactive oxygen species production and proapoptotic caspase-3 activation [77]. The flavonoid antioxidant MESNA is suggested to the application combined with CY to prevent the occurrence of cystitis by neutralizing acrolein and chloroacetaldehyde [78]. However, MESNA is often recommended for high-dose CY (≥10 mg/kg), while the dose of CY in lupus nephritis is far from enough. Thus, the utilization of MESNA is not very common in LN treatment in contrast to cancer therapy.

### 5.5. Carcinogenic Effect

The carcinogenic effect of CY may be related to cumulative dose. If the cumulative dose of oral CY does not exceed 10 g, malignant tumors rarely occur. The incidence of malignant tumors increases with the increased use of CY. The risk of malignant tumor becomes very high when the content of CY exceeds 100 g. Studies have reported that CY can increase the risk of skin cancer, non-Hodgkin lymphoma, and bladder cancer [79]. LN patients treated with oral CY have a higher probability of tumorigenesis than those treated with IV CY during induce remission.

## 6. Conclusion

This review summarizes current knowledge of the use of CY in the treatment of LN. CY is a drug with long-term follow-up and long-term prognosis data. Other drugs take him as the control to judge the effectiveness. Although it has many side effects, CY is still a cheap and easy-to-use drug. With the in-depth study of the mechanism of its side effects, it may be considered to add antioxidants to alleviate the toxic and side effects of CY. Oral administration is an optional treatment method, and intravenous injection has better compliance and prevents patients from taking drugs not on time or taking excessive drugs. Therefore, intravenous injection is more recommended. In the future, the concept and technology of precision medicine may be used to screen the population susceptible to CY-induced remission to alleviate LN through gene diagnosis. Medication regimens will become more reasonable, which will significantly improve the blood concentration of CY, and give full play to the advantageous role of CY in inducing and alleviating LN.

## Figures and Tables

**Table 1:** Use of CY in different guidelines of LN treatment.

Guideline	Kidney Disease Global Outcomes (KDIGO)	American College of Rheumatology (ACR)	The European Alliance of Associations for Rheumatology (EULAR)
Usage	High-dose intravenous CTX regimen (0.75~1.0/m^2^, once a month, treatment for 6 months)		Low-dose intravenous CTX (500 mg, once every 2 weeks, 6 consecutive times, the total amount does not exceed 3 g within 3 months
Side effects	Long-term use of high-dose CTX has resulted in a series of serious adverse reactions, especially irreversible reproductive toxicity		Infection, menstrual disorder, amenorrhea, and bone marrow suppression reduced

**Table 2:** Representative studies of cyclophosphamide treatment compared to MMF or calcineurin inhibitors.

	CY	MMF	Tacrolimus
Common mechanism	Inhibiting lymphocyte propagation (antiproliferation) [9]		
Characteristics of respective mechanisms	Control vascular inflammatory lesions [57].	Inhibits T and B lymphocytes Inhibits lesions of vascular endothelial cells [58].	Inhibits interleukin-10 and affects B lymphocyte function Control the membranous lesions [59].
Efficacy comparison	Improves renal outcomes and have long been considered the gold standard for inducing renal remission and preventing renal flares [43].	The complete and total remission rates are comparable to CY [53].	Respond no inferior than CY and is more efficacious than MMF [56] The effect of reducing urinary protein is stronger (stabilizing podocyte) [60].
Safety comparison	An increased risk of serious infections, myelosuppression, ovarian toxicity, and carcinogenic effect [46].	The risk of serious infections, leukopenia, gastrointestinal response, and ovarian toxicity was lower with MMF than with CY but easy to cause fetal malformation and abortion [53].	No reproductive inhibition and safe to use during pregnancy (better than MMF and CY) [61] The risk of serious infections is lower with tacrolimus than with MMF and CY [61].

## Data Availability

Not Applicable. This is a review without experimental data.

## Additional Points

*Key Summary Points.* (1) Metabolism of cyclophosphamide is modulated by polymorphisms in drug-metabolizing enzymes and combined drugs. (2) The regulation of cellular immunity by cyclophosphamide is both dose-dependent and selective. (3) Intravenous injection of cyclophosphamide is more recommended for better compliance and guarantees patients taking drugs with punctuality and accuracy. (4) It is considered to add antioxidants to alleviate the side effects of cyclophosphamide.
